# Advocating Patient-Centred Research in Ocular Myasthenia Gravis (OMG): A Call for an OMG Research Consortium

**DOI:** 10.3389/fopht.2022.912805

**Published:** 2022-07-12

**Authors:** Sui Hsien Wong

**Affiliations:** ^1^ Department of Neuro-Ophthalmology, Moorfields Eye Hospital National Health Service (NHS) Foundation Trust, London, United Kingdom; ^2^ Department of Ophthalmology, Guy’s and St Thomas’ National Health Service (NHS) Foundation Trust, London, United Kingdom; ^3^ Department of Neurology, Guy’s and St Thomas’ National Health Service (NHS) Foundation Trust, London, United Kingdom; ^4^ King’s College London Faculty of Life Sciences & Medicine, Kings College London, London, United Kingdom; ^5^ Institute of Neurology, University College London, London, United Kingdom

**Keywords:** myastenia gravis, patient–centred care, extraocular muscle, ocular myasthenia gravis (OMG), prognosis

## Introduction

Ocular symptoms in myasthenia gravis (MG) are common, affecting up to 85% of patients with this autoimmune neuromuscular junction disease (1, 2). MG can cause ocular symptoms of diplopia and ptosis, and generalised weakness of limb, bulbar or respiratory muscles. In patients with MG limited to ocular signs and symptoms only, i.e. Ocular Myasthenia Gravis (3, 4), there is a 30-80% risk of converting to Generalised Myasthenia Gravis (GMG) (1, 2, 5, 6).

Traditionally, OMG has been considered a milder version of MG, classified as Class I in the Myasthenia Gravis Foundation of America (MGFA) clinical classification where the consecutive numerical increase from Classes I to IV indicates worsening severity (7).

However, diplopia and ptosis can cause significant disability and impact quality of life (8, 9), which raises the question of whether OMG (MGFA Class I) is truly milder in its impact to patients, compared to mild limb weakness of GMG (MGFA Class II). Therefore, this opinion paper advocates that OMG is best considered a subgroup of MG (10), rather than the mildest subtype of MG, concurring with other researchers in this area (10).

This is also pertinent as there are unique considerations to OMG, some outlined below. These and more, would be research questions best served through concerted collaborations within an OMG research consortium – this paper is a call for such a collegial consortium.

## OMG Rating Scales and Patient Reported Outcome Measures

MG rating scales focus on GMG symptoms and insufficient for monitoring people with OMG (11). To truly support patient-centred care, we need to fully understand the extent of disease impact on our patients (12). An extended ocular version of the Quantitative Myasthenia Gravis (ocular-QMG) rating scale showed promise as a tool for monitoring change of ocular symptoms, in a study demonstrating the effectiveness of steroids, although the ocular-QMG scale was not further validated (13). The Ocular Myasthenia Gravis Rating Scale (the OMGRate) was developed to address this gap (14). The OMGRate comprises of two components: a clinical examination (OMGRate-e) and a patient questionnaire (OMGRate-q). Data from a single-centre of 104 patients (67 males, mean age 55 years, range 18-86), showed good external validity: the examination component of the rating scale, OMGRate-e, had good correlation with the MGC (r=0.64, 95% confidence intervals [CI] 0.54-0.74, p<.0001); good correlation between the OMGRate and MG-QOL15 (r=0.68, 95% CI 0.60-0.77, p<.0001). As the cohort studied was from a single centre, the next step is to validate the OMGRate in a multicentre study.

Alongside this, patient report outcome measures (PROMs) are also needed for patient-centred research, as PROMS measure the impact of disease and treatments without intervening interpretation from clinicians and researchers (15). For example, the degree of ocular deviation or measurement of ptosis is not as important to the patient, compared to the functional impact of this on their daily activities. The use of PROM in MG has been shown to be effective and facilitates delivery of tele-healthcare (16). The questionnaire portion of the OMGRate (the OMGRate-q) has shown good external validation and therefore potential to be validated as a standalone PROM for OMG (14). A high-quality PROM for OMG is critical for patient-centred research trials comparing OMG treatments, particularly with new therapies in the horizon (17).

## Monitoring Care in Clinic

An important aspect of care in OMG is to monitor for the development of GMG, including limb, bulbar and respiratory weakness. The respiratory function assessment in MG traditionally uses a spirometer to measure the forced vital capacity (FVC), and a reduction of FVC from standing to supine positions could indicate diaphragmatic weakness (18). Unfortunately, spirometry is aerosol generating, limiting its use since the COVID-19 pandemic. The single breath count (SBC) can be a surrogate measure of vital capacity in GMG (19), useful for screening for exacerbations of MG (20), and in OMG may show a decrease in SBC prior to onset of respiratory symptoms (21). An alternative, non-aerosol generating method of monitoring respiratory function test is the sniff nasal inspiratory pressure (SNIP) (22), although research to validate this in OMG is needed.

## Improving Diagnostic Yield

One of the potential difficulties experienced by people with OMG, is the delay in diagnosis, particularly in seronegative patients who comprise 40-50% of all OMG (4, 5). Approximately 50% of people with OMG have antibodies against the acetylcholine receptor (AChR) (4, 5). A small number have the antibodies against the muscle specific kinase (MuSK) or the low-density lipoprotein receptor related protein 4 (LRP4) (4). The serological diagnostic yield can be improved with cell-based assays, where the clustered AChR receptor antibodies can be detected in up to 25% of seronegative OMG (23). Therefore, a proportion of patients are still seronegative, and concerted effort to discover antibodies in these patients will aid clinical diagnosis.

Neurophysiology with repetitive nerve stimulation have limited sensitivity in OMG (4). Single fibre electromyography (SFEMG) of frontalis or orbicularis oculi can improve the sensitivity rate, but can also be abnormal in other non-MG conditions (4, 17). SFEMG of EOM muscle has been reported (24), but not widely adopted. More recently, the ocular vestibular evoked myogenic potentials (oVEMP) have shown promise as a diagnostic tool in MG. In a study of 27 patients with MG (13 OMG, 14 GMG), oVEMP had an 89% sensitivity and 64% specificity for MG (25, 26). The oVEMP is a promising novel diagnostic tool and merits further research into its clinical utility, particularly in seronegative OMG where diagnosis is arguably more difficult.

## Why Ocular Muscles Only? Clinical Phenotyping and Extraocular Muscles Research

An interesting unanswered question is why some patients with OMG have a predominantly ‘ophthalmoplegic’ phenotype with large ocular deviations, whereas others have minimal extraocular muscle weakness with significant variability in signs and symptoms. Clinical phenotyping of OMG by clinical examination and serology may provide further insights into its pathophysiology, particularly alongside the study of extraocular muscles (EOMs).

EOMs are unique and differ from skeletal muscles, possessing six different muscle fibre types, compared to four in skeletal muscles, of different size, contractile speeds, & fatigue resistance (27). The reasons for these differences are not fully understood, but likely due to the unique demands of eye movements for speed, endurance of static holds, & precision.

**Figure 1:**
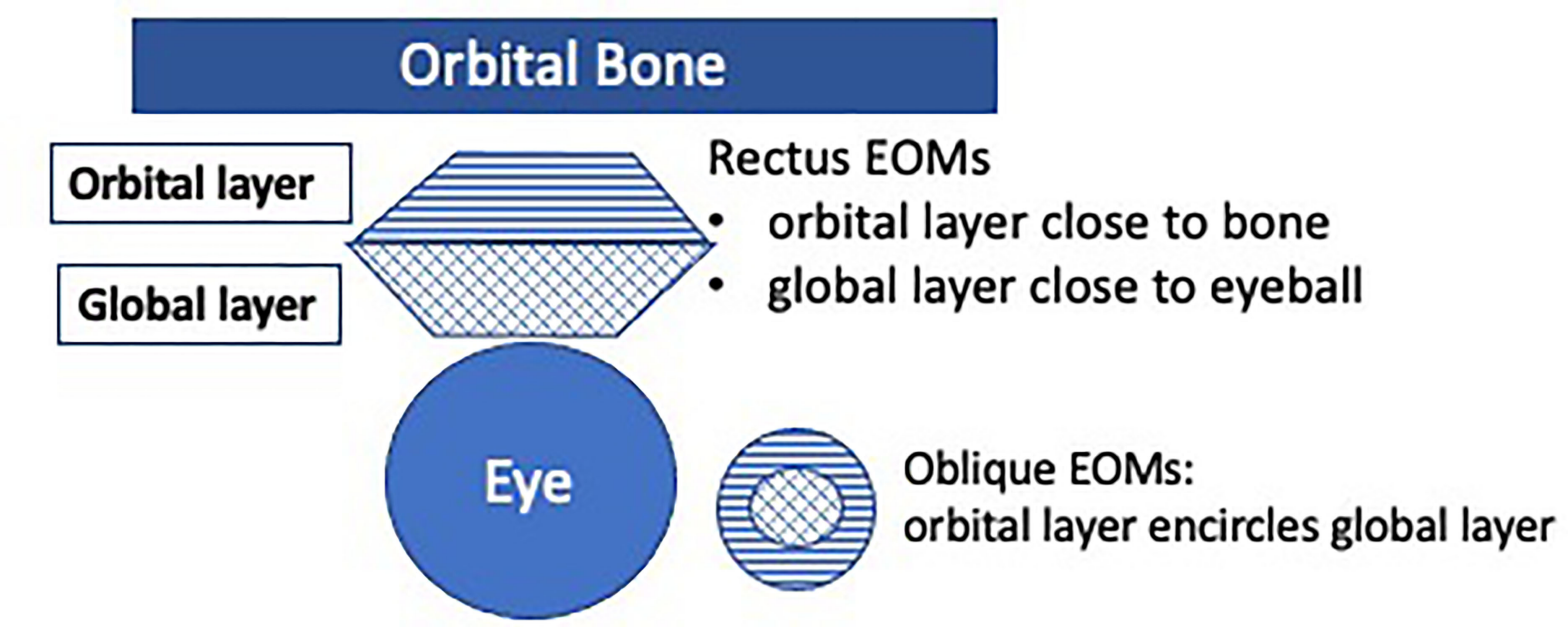
The extraocular muscles (EOMs) are divided into two layers: the orbital and the global layers. The orbital layer initiates movement, and the global layer activates later. Orbital layers consist of 20% multiply innervated fibres (MIF), and 80% fast-twitch singly innervated fibres (SIF). Global layers consist of 10% slow-twitch MIF and 90% fast-twitch SIF (including red, intermediate and white muscle fibres) (27).

EOMs are susceptible in MG due to reduced folding of the postsynaptic membrane at the neuro- muscular junction (28), deficiency in complement inhibitory proteins (29), and a high physiological demand for sustained and precise ocular alignment (28). EOMs are also divided into global and the orbital layers (see **Figure 1**), with functional differences as reflected by different fibre types in these respective layers, e.g. in initiating eye movement versus gaze holding (27, 30–32). A hypothesis of interest to study in OMG, is whether the orbital and global layers are preferentially affected in the different OMG clinical phenotypes.

Gene expression studies are a promising area of research for OMG, as they can reveal functional differences in the EOM layers (33, 34). In contrast to anatomical studies which look at the morphology of the tissue, RNA expression can show differences in functions of cells of similar morphology, and can also elucidate effects of epigenetics or chromatin control in the way genes are expressed (35).

Oculography studies to objectively measure eye movements in OMG can shed further insight into its pathophysiology. Cogan and colleagues demonstrated that in MG with severe ophthalmoplegia, there was relative sparing of fast twitch fibres when tonic muscle fibres responsible for maintenance of eccentric gaze hold were severely affected (36). They also showed that in some MG patients, small amplitude saccades were hypermetric and had high velocities, appearing as twitch/quiver movements characteristic of MG (36, 37). Barton and Sharpe showed that saccades of MG patients had more variability in the initial and fatigue periods compared to those without MG, concluding that saccadic jitter may be a useful diagnostic sign in 42% of myasthenic saccadic analysis (38). Additionally, increased microsaccadic movements in OMG compared to healthy controls may suggest frequent ‘recalibration’ to maintain gaze hold (39).

Another promising area of research is the use of magnetic resonance imaging (MRI) to evaluate the EOM in MG. Recent research techniques showing fat replacement or atrophy in MG can help elucidate the disease pathophysiology (40, 41). Using these techniques as part of the clinical phenotyping of OMG can be illuminating.

## The Risk of Secondary Generalised Myasthenia Gravis

Last but not least, the risk of SGMG is another important area for further research. The reported risk of SGMG has varied widely, ranging from 30-80% (1, 2, 5, 6). Whether the risk of SGMG can be modulated by immunosuppression or thymectomy remains controversial (5). To address this, a large randomised controlled trial of 304 newly diagnosed OMG will need to be recruited (42), and such an endeavour can only be achieved through a large multicentre study. However, the sample size needed may be less if we can selectively recruit patients at high risk of SGMG. Our earlier work has showed by proof-of-principle that a ‘risk of generalization’ (ROG) score can be created, allowing us to stratify patients into high or low risk of SGMG (42). This study was based on a retrospective cohort. Data analysis to further develop the ROG score with a prospective cohort is currently in progress (43).

There are a number of high-quality MG registries (44, 45), and work is also needed alongside this for an OMG registry with clinical phenotyping of OMG (43). Such a registry will prepare the groundwork for future treatment trials, supporting successful participant recruitment. Such registries will also support development of biomarker studies on predicting the risk of SGMG (46), and the search for antibodies in seronegative patients. Severity of OMG can be a risk for SGMG (47), and a multicentre OMG registry, that includes high quality PROM and rating scales, can support the ongoing development and refinement of a robust ROG score. A robust ROG score will also allow us to better counsel patients on their risk of SGMG, as part of delivering patient-centred care.

## Conclusions

With rare conditions such as OMG where research funding is relatively limited, a concerted, cohesive and collaborative effort is the way forward, for the benefit of patients who are most affected by this condition. An OMG research consortium that pulls together researchers in the field and our respective strengths and expertise, will benefit patients, enable high quality patient-centred research that translates to patient-centred care.

## Author Contributions

The author confirms being the sole contributor of this work and has approved it for publication.

## Conflict of Interest

The author declares that the research was conducted in the absence of any commercial or financial relationships that could be construed as a potential conflict of interest.

## Publisher’s Note

All claims expressed in this article are solely those of the authors and do not necessarily represent those of their affiliated organizations, or those of the publisher, the editors and the reviewers. Any product that may be evaluated in this article, or claim that may be made by its manufacturer, is not guaranteed or endorsed by the publisher.
